# Warfarin‐induced spontaneous iliopsoas hematoma — An unusual complication

**DOI:** 10.1002/ccr3.1665

**Published:** 2018-06-22

**Authors:** Babikir Kheiri, Mohammed Al Salihi, David Maldonado, Rasha Nakhleh, Ghassan Bachuwa

**Affiliations:** ^1^ Internal Medicine Department Hurley Medical Center/Michigan State University Flint MI USA

**Keywords:** iliopsoas hematoma, spontaneous hematoma, warfarin

## Abstract

Warfarin is a commonly prescribed anticoagulant with a narrow therapeutic window and high potential for serious complications. Spontaneous psoas hematoma is a rare complication of warfarin which can result in significant neurological deficits.

## QUIZ QUESTION: WHAT IS THE DIAGNOSIS AND THE MANAGEMENT?

**Figure 1:**
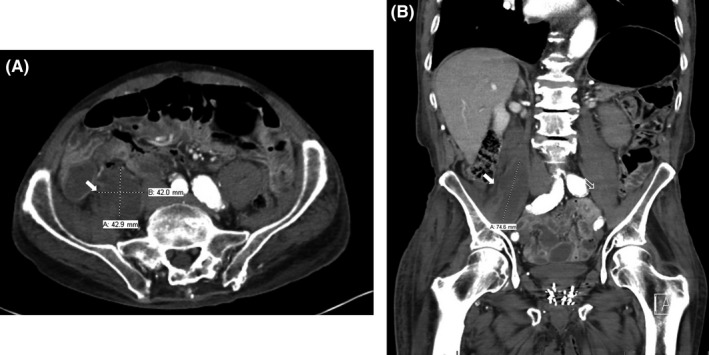
Coronal (A) and axial (B) section of the CT abdomen showing right psoas hematoma (filled arrows) vs normal structures (unfilled arrow)

A 91‐year‐old gentleman with a medical history of atrial fibrillation on warfarin, brought to the hospital for a 1‐week history of worsening right thigh pain, radiating to his right lower leg with loss of ambulation secondary to his increasing weakness. He denies any history of falls/trauma. On examination, he was hemodynamically stable. Neurological examination was significant for wasting of the right quadriceps, reduced power (1/5) at right hip flexors, reduced sensations at right L2‐4 distribution, and right patellar hyporeflexia. Initial investigations showed a supra‐therapeutic INR at 5.31. A contrast‐enhanced CT scan showed a 4.3 × 4.2 × 7.5 cm right psoas hematoma with no evidence of active bleeding (see Figure 1). His neurological deficits were attributed to mechanical compression of the right upper lumbar plexus. Therefore, warfarin was initially reversed with vitamin K and he underwent an unsuccessful CT‐guided hematoma drainage. A decision was made for conservative management given his stable hemodynamic status without worsening symptoms or radiographical findings. After undergoing extensive physical therapy, he began to ambulate and recover slowly.

Spontaneous iliopsoas hematoma is a rare complication of warfarin therapy.^1^ The management depends on the patient's hemodynamic status, comorbidities, and the presence of active bleeding.^1^ Treatment strategies could include surgical decompression, intervention radiology, and/or conservative approaches with cessation/reversal of warfarin along with physical therapy.^1^, ^2^ Physicians should be aware of such complication in any patients presenting with lower limb symptoms to avoid catastrophic permanent neurological deficits.

## CONFLICT OF INTEREST

None declared.

## AUTHORSHIP

BK: designed, planned, wrote the manuscript, and did the literature review. MA:‎ designed, planned, and revised the manuscript. DM: designed, planned, and revised the manuscript. RN: designed, planned, and revised the manuscript. GB: designed, planned, and revised the manuscript.
